# Exploring perceived barriers and attitudes in young adults towards antidepressant pharmacotherapy, including the implementation of pharmacogenetic testing to optimize prescription practices

**DOI:** 10.3389/fphar.2024.1526101

**Published:** 2025-01-03

**Authors:** Bradley Roberts, Zahra Cooper, Georgia Landery, Susanne Stanley, Bernadette T. Majda, Khan R. L. Collins, P. Anthony Akkari, Sean D. Hood, Jennifer Rodger

**Affiliations:** ^1^ Perron Institute for Neurological and Translational Science, Nedlands, WA, Australia; ^2^ School of Biological Sciences, The University of Western Australia, Crawley, WA, Australia; ^3^ School of Biomedical Sciences, The University of Western Australia, Crawley, WA, Australia; ^4^ Division of Psychiatry, School of Medicine, The University of Western Australia, Crawley, WA, Australia; ^5^ Curtin Medical School, Curtin University, Bentley, WA, Australia; ^6^ North Metropolitan Health Service, Western Australian Department of Health, Nedlands, WA, Australia; ^7^ School of Human Sciences, The University of Western Australia, Crawley, WA, Australia; ^8^ Centre for Molecular Medicine and Innovative Therapeutics, Murdoch University, Murdoch, WA, Australia; ^9^ Division of Neurology, Duke University Medical Centre, Duke University, Durham, NC, United States

**Keywords:** pharmacogenetics, antidepressant pharmacotherapy, depression and anxiety, youth, young adults, community perspective, clinical implementation

## Abstract

**Introduction:**

The field of pharmacogenetics (PGx) is experiencing significant growth, with increasing evidence to support its application in psychiatric care, suggesting its potential to personalize treatment plans, optimize medication efficacy, and reduce adverse drug reactions. However, the perceived utility and practicability of PGx for psychiatric treatment in youth remains underexplored. This study investigated perceived barriers and attitudes in Australian young adults towards the implementation of PGx testing to guide antidepressant treatment in primary care.

**Methods:**

Semi-structured focus groups and interviews were conducted with 17 participants aged between 18 and 24 years. These sessions were recorded and transcribed before thematic analysis was used to identify collective themes.

**Results:**

Three key themes were identified, including attitudes towards the medication prescription process, concerns and attitudes towards PGx testing, and perceived barriers to its clinical implementation. Although PGx testing was positively perceived by most participants, all participants shared concerns about PGx testing. Participants voiced concerns about the financial impact of PGx testing, the potential for treatment delays, and the accuracy of PGx testing in guiding antidepressant treatment. Additionally, participants noted that the low awareness and willingness of general practitioners to incorporate PGx testing into routine practice could hinder successful clinical implementation.

**Discussion:**

Prior to the implementation of PGx testing into Australian primary practices, it is essential to acknowledge patient perspectives and ensure that clinical practices remain patient-focused. This study highlights important considerations for integrating PGx testing into antidepressant pharmacotherapy and emphasizes the need for future research to address and mitigate the perceived barriers of young adults.

## 1 Introduction

Over one in five Australian young adults under the age of 25 experience symptoms of depression and/or anxiety (Australian Institute of Health and Welfare, 2021b; Lawrence et al., 2015). Whilst antidepressants are often considered the first-line pharmacotherapy for these conditions (Malhi et al., 2015; Santarsieri and Schwartz, 2015), these medications have consistently produced poor results in youth patients (Strawn et al., 2023a; Bridge et al., 2007; March et al., 2004; Weihs et al., 2018; Emslie et al., 2014; Durgam et al., 2018; Le Noury et al., 2015; Davey et al., 2019; Safer and Zito, 2019; Spielmans and Gerwig, 2014; Cipriani et al., 2016), with over one-third of youth treated with antidepressant pharmacotherapy showing little to no response to their antidepressant treatment (Kennard et al., 2009). Where patients fail to respond to an adequate antidepressant trial, another is often prescribed in a trial-and-error manner (Leuchter et al., 2009). However, the likelihood of future response and remission with antidepressant pharmacotherapy decreases dramatically with subsequent antidepressant trials (Rush et al., 2006; Gaynes et al., 2009). Similarly, the risk of severe medication-related side effects, which may significantly impact quality of life, increases with each consecutively prescribed antidepressant. It is therefore crucial to ensure that antidepressants are prescribed optimally from the initial treatment.

The Australian prescription rate of antidepressants has risen by ∼33% over the past 7 years (Australian Institute of Health and Welfare, 2023; Australian Institute of Health and Welfare, 2022; Australian Institute of Health and Welfare, 2021a; Australian Institute of Health and Welfare, 2020; Australian Institute of Health and Welfare, 2019; Australian Institute of Health and Welfare, 2018), with over 33 million antidepressants prescribed in 2023 alone (Australian Institute of Health and Welfare, 2023). In recent years, antidepressant use in young Australians under 25 years increased by 25% and new antidepressant prescriptions to those not previously prescribed, rose by over 33% (de Oliveira Costa et al., 2023). Given that over 85% of antidepressants in Australia are prescribed by general practitioners (GPs) (Australian Institute of Health and Welfare, 2023), there is a considerable need to reanalyze primary mental healthcare practices to improve the effectiveness of antidepressant pharmacotherapy and ensure that youth receive optimal care (Le et al., 2021).

Pharmacogenetics (PGx), the study of how interindividual genetic variation affects a person’s response to medication, has been suggested to aid clinicians in the medication selection process, personalizing a patient’s prescription portfolio (Bousman et al., 2023a; Strawn et al., 2023b). Numerous studies have shown that PGx-guided pharmacotherapy improves the therapeutic effect of antidepressant treatment, decreases the likelihood of adverse drug reactions and medication-related side effects, and increases patient medication adherence (Hall-Flavin et al., 2012; Hall-Flavin et al., 2013; Greden et al., 2019; Oslin et al., 2022; Han et al., 2018; Vos et al., 2023; Pérez et al., 2017; Bradley et al., 2018; Brown et al., 2022; Bousman et al., 2019; Rosenblat et al., 2018; Wang et al., 2023; Olson et al., 2017; Nooraeen et al., 2024; Skokou et al., 2024; Platona et al., 2024; Xu et al., 2024). PGx has also been suggested to have particular relevance in the treatment of depression and anxiety in young people due to the low efficacy rate, heightened risk profile, and low medication adherence rates observed in youth compared to adults (Gast and Mathes, 2019). Though support for PGx is still emerging (Nooraeen et al., 2024; Ariefdjohan et al., 2021; Aldrich et al., 2019; Poweleit et al., 2019; Namerow et al., 2022; Voort et al., 2022), the rapidly expanding capabilities for PGx-guided therapeutics and the developing guidelines by the Clinical Pharmacogenetic Implementation Consortium (CPIC) offer actionable recommendations for commonly prescribed antidepressants, such as selective serotonin reuptake inhibitors and tricyclic antidepressants (Hicks et al., 2017; Bousman et al., 2023b). These CPIC guidelines provide genotype-based dosing recommendations and may inform clinical decision-making, addressing efficacy and tolerability concerns. Such advancements could potentially address these challenges, facilitating wider implementation for young people with depression and anxiety (Roberts et al., 2023).

To date, the uptake of PGx-guided pharmacotherapy in practice in Australia has been slow, with previous studies bringing to light several barriers impeding clinical integration (Jameson et al., 2021; Pinzón-Espinosa et al., 2022; Virelli et al., 2021). Previous research aimed to understand the perspectives of key stakeholders, investigating attitudes towards PGx-guided mental healthcare among various groups, such as adult patients (Kastrinos et al., 2021; Liko et al., 2020; McCarthy et al., 2021; Slomp et al., 2022), practitioners and healthcare specialists (Slomp et al., 2022; Chan et al., 2017; Shishko et al., 2015; Aboelbaha et al., 2023; Vest et al., 2020; Lanktree et al., 2014; Thompson et al., 2015; Dunbar et al., 2012; Goodspeed et al., 2019; Laplace et al., 2021; Sperber et al., 2024), and even youth psychiatrists (Soda et al., 2023; Liko et al., 2021; Jessel et al., 2022). However, little research has evaluated the perspectives of younger patient populations, and those that have, did so only after PGx testing had taken place (Stancil et al., 2021).

Young people are increasingly seeking more involvement in their healthcare decision making (Fleary and Joseph, 2020; Simmons et al., 2011). Therefore, it is important to understand their perspectives and views on clinical interventions such as PGx to ensure that their needs and expectations are met during its implementation into clinical use (Subasri et al., 2023). Given the limited research on youth perspectives, this study aimed to understand young people’s concerns surrounding antidepressant treatment, their attitudes and perspectives on PGx testing in antidepressant pharmacotherapy, and the perceived barriers that may inhibit Australian youth from accessing PGx testing in primary mental healthcare.

## 2 Materials and methods

### 2.1 Study aim and design

A qualitative approach utilizing focus groups and interviews was employed to gain a deeper understanding of youth perspectives of PGx-guided antidepressant pharmacotherapy. For the purpose of this study, the term “youth” will refer to young adults aged 18–24 years, unless otherwise specified. This methodology was selected to gather rich, detailed data, providing valuable insights into the nuanced and complex views of participants, often challenging to quantify. Both inductive and deductive coding was used to explore organically emerging themes and those guided by our principal framework.

This study is part of a larger co-design research project consisting of two consecutive stages: (1) a) exploratory focus groups and interviews with young adults with a lived experience of depression and/or anxiety, and b) case study discussions with GPs with an interest in treating youth mental health, and (2) a pilot randomized controlled trial evaluating the effectiveness of PGx-guided antidepressant pharmacotherapy in young people. This iterative design ensures the findings of the focus groups and interviews inform the development of the outcome measures for the pilot randomized controlled trial. This paper will report on the findings obtained from the focus groups and interviews with young adults.

### 2.2 Participant recruitment

Purposive sampling was used to recruit participants for focus group and interview discussions. Eligibility criteria included (1) age between the ages of 18 and 24 years inclusive, (2) self-reported current or past antidepressant pharmacotherapy, and (3) fluency in written and spoken English sufficient to provide informed consent. Individuals who were unable to give informed consent due to cognitive or linguistic reasons were excluded from participation. Recruitment methods included advertisements on social media, fliers on university campuses, and snowballing sampling through word-of-mouth.

This study was approved by the Government of Western Australia, Department of Health (RGS0000005473) and endorsed by the University of Western Australia (2023/ET000209). All eligible participants expressing interest were emailed study information letters. Informed consent was obtained from all participants prior to taking part in a focus group or interview discussion along with completion of a participant demographic form. Reminder emails were sent 1 week prior to the session and repeated 24 h before the scheduled focus group or interview session. All sessions were held in a private meeting room at the Perron Institute for Neurological and Translational Science, Perth.

### 2.3 Focus group and interview discussions

Semi-structured focus groups and interviews were conducted to explore the attitudes and perceptions of young people toward PGx testing in antidepressant pharmacotherapy. Where feasible, participant scheduling prioritized focus groups of 2–4 participants to facilitate interactive discussions, allowing for a broader range of perspectives. Where focus groups were not possible due to participant preference or availability, individual interviews were conducted. All focus groups and interviews were moderated by ZC and BR and lasted approximately 60 min. Recruitment continued until thematic saturation was attained.

Facilitators followed the principal guide sequentially but allowed flexibility for participants to lead the conversation and contribute to each question. Facilitated prompts were used when discussion waned. Questions in the principal guide were constructed from prior literary review and the aid of community members from the Western Australian Health Translation Network’s Consumer and Community Involvement Program to ensure that the questions asked were targeted and framed appropriately for understanding by the youth demographic (Roberts et al., 2023). Each session included the eight questions presented in Table 1.

**TABLE 1:** Principal questions for focus groups and interviews.

1. What is your experience in getting treatment for depression and/or anxiety?
2. What are your questions or concerns when starting a new medicine?
3. What do you think affects a person’s response to new medication?
4. What do you see as advantages of PGx testing in depression and/or anxiety?
5. What concerns would you have if you were offered DNA testing to select the right medication for your depression or anxiety?
6. Do you foresee any barriers to getting PGx testing for the treatment of depression and/or anxiety?
7. In general, do you find that the information that you received from healthcare professionals (such as GPs) about your anxiety and/or depression and/or medication is adequate?
8. How can GPs offer PGx testing in the treatment of mental health conditions? How can GPs improve the information you receive about your treatment plan?

### 2.4 Data collection

All focus groups and interviews were audio recorded and transcribed using an orthographic style to maintain the accuracy and integrity of the data. Transcripts were edited to preserve the anonymity of participants, removing names, and replacing them with unique identifiers (i.e., FG1P1).

### 2.5 Data analysis

Ritchie and Spencer’s Thematic Analysis Framework was used to analyze each focus group and interview session (Ritchie and Spencer, 1994). Given the semi-structured nature of the sessions allowing for participant-led discussions, novel themes were also constructed using reflexive thematic analysis techniques as outlined by Braun and Clarke (2006). More information on the thematic analysis process used in this study is provided in Supplementary Material S1.

First-pass coding was conducted by BR using MS Excel to identify broad-scope ideas expressed by participants in each focus group or interview. This process helped determine whether thematic data saturation had been reached. Second-pass coding grouped these codes into novel or predetermined themes. The data was then independently coded by GL. Differing codes were moderated and finalized by ZC.

## 3 Results

### 3.1 Participant demographics

Five focus groups and four one-on-one interviews were conducted with focus groups ranging 2-3 participants per group session. A total of 32 young people expressed interest to partake in the current study. Of these, 17 participants met eligibility requirements and attended and participated in either a focus group or interview discussion. Most participants were female (*n* = 12, 70.6%) with a post-high school education (*n* = 12, 70.6%); approximately 52.3% (*n* = 9) of the sample were born outside of Australia. Full demographic details of all participants are provided in Table 2.

**TABLE 2:** Participant demographic data.

Characteristic	*n* (%^a^)
Sex (*n* = 17)	
Female	12 (71)
Male	4 (23)
Non-Binary/Gender Diverse	1 (6)
Level of Education (*n* = 17)	
Completed High School	5 (29)
Completing Undergraduate Degree	7 (41)
Completed Undergraduate Degree	3 (18)
Completing Postgraduate Degree	2 (12)
Place of Birth (*n* = 17)	
Within Australia	8 (47)
Overseas	9 (53)
Both Parents Born Outside Australia (*n* = 16)	
No	8 (50)
Yes	8 (50)
Language Other Than English Spoken at Home (*n* = 17)	
No	10 (59)
Yes	7 (41)

^a^ Percentages have been rounded to the whole number. Table columns therefore approximate 100%.

### 3.2 Findings

Themes from the focus groups and interviews included statements related to youth experiences and perceptions on current antidepressant prescription procedures and their thoughts and concerns regarding the implementation of PGx testing to inform antidepressant pharmacotherapy. This paper reports on arising themes specifically related to (Australian Institute of Health and Welfare, 2021b): concerns when starting antidepressant pharmacotherapy (Lawrence et al., 2015), perspectives and concerns surrounding pharmacogenetic testing, and (Malhi et al., 2015) youth-perceived barriers to pharmacogenetic implementation, as these themes relate to the implementation of PGx into clinical practice for youth depression. Key findings are summarized in Table 3.

**TABLE 3:** Summary of the thematic results from youth participants relating to their perspectives on the implementation to PGx integration in primary psychiatric care.

Theme	Findings
Concerns when starting antidepressant pharmacotherapy	
Lack of involvement in the treatment process	• Participant was not involved in the medication selection process • Prescription process was not personalized to the individual and was made based on general medication guidelines • Participant expressed the desire for more personalized an streamlined treatment • Participant expressed the desire to be included in the medication selection process • No discussion about medication and the possible side effects that may present was had between patient and clinician before antidepressants were prescribed • Participant expressed the desire to hear about alternatives to pharmacotherapy before starting their antidepressant prescription
Treatment effectiveness and tolerability	• Presentation of medication-related side effects • Dependency on antidepressant medication • Efficacy of drug to achieve a therapeutic response • Trial-and-error process of finding the right medication • Effects on behavioral traits and personality
Perspectives and concerns surrounding pharmacogenetic testing	
Financial impact	• Cost concern for PGx testing • Overestimation of PGx testing costs • Financial and familial concerns specific to young people • The costs associated with frequent visits to GPs • Appropriate estimation of PGx testing costs
Treatment delay	• Delay in care with PGx testing • No concern over wait time • Overestimation of PGx testing wait time • Skepticism around the logistics of PGx testing • Over a 1-week wait is harmful • Mental healthcare system wait times are long anyway so waiting
Testing accuracy	• Efficacy of PGx testing
Invasiveness	• Physical invasiveness of PGx testing • Invasiveness on data privacy
Youth-perceived barriers to pharmacogenetic testing implementation	
Knowledge and willingness of GPs to adopt PGx testing	• Lack of GP understanding about PGx testing and its value in clinical practice • GPs may be unwilling to adopt and implement the recommendations provided by PGx testing
Affordability	• High costs that are currently uninsured by Medicare or private health insurance
Lack of awareness	• Lack of awareness of PGx in youth • Lack of community education around genetic testing
Limited accessibility	• Lack of easy access to testing and information on PGx testing • People in rural regions may find it difficult to get access to PGx testing kits

#### 3.2.1 Concerns when starting antidepressant pharmacotherapy

Youth participants were prompted to share their experiences when receiving antidepressant medication, with all participants sharing at least one major concern they had during this treatment process. Two commonly recurring subthemes arose in all focus groups and interview sessions: 1) lack of involvement in the treatment process, and 2) effectiveness and tolerability.

##### 3.2.1.1 Lack of involvement in the treatment process

Many participants reported a significant concern regarding their involvement in the medication selection process. Specifically, they felt excluded from treatment decisions leading to feelings of disempowerment and frustration, and ultimately, losing trust in their clinician’s treatment decisions.

*“I told you my preference and you just completely disregarded it. So, I got put on Prozac and my health took a plunge. Like a really, really bad plunge.”* [FG2P2]

Furthermore, participants frequently highlighted the impersonal nature of the prescription process, sharing that treatments were often prescribed based on general guidelines rather than investigating individualized needs.

*“…the way that they prescribe, they had a flip book of medications, and they were like, “yeah, do you want that one?” Like, do I look like I’m here to shop? I’m here to talk about my health.”* [FG2P2]

Participants shared the desire to be included in the decision-making process, with strong preferences for a more collaborative approach with healthcare providers and conversations to determine shared expectations and treatment goals. Participants felt that these crucial conversations were often neglected, leaving them unprepared and ill-informed when it came to their antidepressant prescription and mental health treatment.

*“Like, they do not really talk to you that much about your background it’s just like, okay, what medication can we try next. So that was not working, let’s try another one. And that’s mainly what my experience consisted of.”* [FG2P3]

Participants also discussed their eagerness to engage in conversations regarding their treatment options, including alternative medications and non-pharmacological interventions, allowing them to be better informed and have agency in their treatment.

*“Whenever I go to the GP, they’re like, “oh yeah. Just have your medication. Okay. Goodbye.” I did not know any of the other options*. *So, I would have liked to know, like choices or other things that I could have had.”* [FG2P1]

##### 3.2.1.2 Treatment effectiveness and tolerability

Almost all participants reported having experienced some form of medication-related side effects, with many participants anxious about future occurrences and the long-term consequences of antidepressants.

*“…those 2–3* *weeks post having started medication was the worst two or three weeks I’ve ever experienced*. *I was shaking the whole time, did not sleep for like weeks*. *It got worse before it got better in terms of the depression symptoms.”* [FG1P1]

Coupled with these experiences, numerous participants also experienced being on antidepressants that had not provided any therapeutic benefit, creating an apprehension for future prescriptions and an expectation that these future treatments would also fail. This uncertainty contributed to the hesitation in starting or continuing with antidepressant pharmacotherapy, with over half the participants from the focus group and interview sessions having not responded to at least one antidepressant medication.

Due to these experiences, participants frequently highlighted the challenge of finding an effective medication, emphasizing the exhaustive nature of the trial-and-error process often required to achieve a therapeutic response. Frustrations with the time-consuming and sometimes discouraging process of trying multiple medications along with the uncertainty about whether any given medication would be effective, often led to participants to cease their treatment without telling their treating clinician.

*“I was on and off medication for 4* *years. I kind of just stopped it because I did not see any effects and it just seemed like the GP was just too busy and just disinterested because you only get your 10* *min. So, I kind of just stopped that by myself.”* [FG7P1]

Lastly, several participants feared becoming reliant on their medication to maintain their mental health. This fear was compounded by worries about changes in behavioral traits and personality, with participants questioning whether the medication would alter their identity, dampening aspects of their personality or changing their behavior in undesirable ways.

*“…but then I feel like now I’m like starting to want to get off antidepressants because I feel like I do not even remember what I was like before I went on antidepressants and like, I feel like I’ve changed a lot since then.”* [FG7P2]

#### 3.2.2 Perspectives and concerns surrounding pharmacogenetic testing

Participants generally viewed PGx testing as a beneficial tool for informing antidepressant prescriptions, recognizing that genetic variability can impact drug metabolism and response. The general consensus was that PGx information could be used to optimize and personalize antidepressant prescriptions.

*“If [PGx testing] was available to me, I would probably go to that in the first instance, just because then I know that it’s, at least somewhat customized to my body and I know that it’s probably going to work. I found it quite a traumatic process of finding which medication works. And I know this would save time, a lot of money, a lot of stress and then not going through those side effects. And also, if you’re able to change the dose to however your body processes medication, you kind of already know where you should be, and it’s only like little minor tweaks that you probably do to the dose as opposed to changing like a whole medication or adding new medications.”* [FG7P3]

Despite this positivity, participants shared several concerns they would wish to see addressed prior to PGx use.

##### 3.2.2.1 Financial impact

Concerns predominantly centered around the costs associated with PGx testing, with participants often perceiving PGx as a financial burden involving frequent visits to the GP to engage with testing processes.

*“[Cost] is the main issue. Whether you are doing the test or whether you’re going to the doctor, both are expensive. So, yeah, cost and the financial burden is, like, in every aspect.”* [FG9P1]

Several participants grossly overestimated the expense of PGx testing, believing it to be prohibitively high, whereas those who understood the costs involved, recognized that 200 AUD would be a reasonable upper limit for their use.

*“If I thought it was going to really do well for me, I’d probably be okay with maybe $200. Yeah, but like anything more than that and I would ask, can I afford this*.*”* [FG6P1]

For a few participants, financial anxieties were compounded by familial concerns, with participants worrying that, as a young person reliant on the financial support of family members, the cost of PGx testing would not be favored by parents when weighed against other medical expenditures.

##### 3.2.2.2 Treatment delay

Mixed views were shared regarding the potential for PGx testing processes to delay treatment. Whilst some participants perceived any wait time for test results as acceptable if it led to more effective treatment, others were concerned that the delay between sample collection and receiving results would postpone the start of their treatment.

*“If you need something right away and want to start taking medication, it might take longer for all the things in this process; for them to do the swab, then to send it out, then to have them analyze it and then give the results back and then get the results to you, it might take a while longer.”* [FG5P1]

Commonly, participants concerned about treatment delays due to PGx testing often overestimated the duration of the testing processes, with some expecting results and medication initiation to take over a month. When focus group and interview moderators explained that the average wait time for testing in Australia was 10 business days (Forbes et al., 2023), these concerns were often alleviated, though some participants stated that more than 1 week is an unacceptable time to wait for PGx results and treatment.

*“If you could be prescribed something a little bit less for a little bit of relief whilst waiting for testing results, you could probably go like several weeks, maybe up to a month. But if you’re on absolutely nothing, probably not more than a week.”* [FG6P1]

##### 3.2.2.3 Testing accuracy

Having experienced failures with prior medications, many participants were concerned about the ability of PGx testing to effectively inform on a medication that would be more appropriate for them and that may more effectively manage their depressive and/or anxiety symptoms.

*“I am a bit dubious about the results. Like it seems so left of field that somebody can take a swab from my cheek, and it will tell me that they know the answers to my, you know, my biggest fears.”* [FG1P1]

##### 3.2.2.4 Invasiveness

Prior to further understanding of what was involved in the testing process, participants believed that PGx testing was a demanding process for mental health patients.

*“I think personally, anything related to hospitals and just going in to test and get blood tests, it’s really yeah, the whole thing is scary for me, personally.”* [FG4P2]

Data-wise, participants voiced their worries about the security and confidentiality of their genetic information. There was a pervasive anxiety that sensitive genetic data could be misused or accessed by unauthorized parties, leading to potential discrimination in future employment or insurance.

#### 3.2.3 Youth-perceived barriers to pharmacogenetic testing implementation

Participants considered the concerns of the broader youth population and suggested factors likely inhibiting the broader use of PGx testing in primary mental healthcare.

##### 3.2.3.1 Knowledge and willingness of GPs to adopt PGx testing

Participants perceived that the understanding and willingness for GPs to integrate PGx testing would stand as a significant barrier to community access. To youth, the perceived lack of understanding meant that GPs would be unfamiliar with how to prescribe medication from patient genotypes and metabolic phenotypes, limiting the value of PGx in primary practice. Furthermore, many participants felt that GPs were not familiar with or adequately informed about how PGx testing could be used in personalized pharmacotherapies and would therefore choose against adopting PGx processes into their routine practice.

*“I think even some professionals may not believe in the science of it. I had the test done and took it to my doctor and she was like, “oh well, this does not really mean anything.” So yeah, I do not know whether there’s some professionals that do not really believe in its validity still.”* [FG4P1]

A consensus amongst participants was that if PGx testing was to succeed in integrating into the mental healthcare environment, it would be crucial that GPs are not only aware of PGx testing as an option for informed prescriptive practices, but that GPs also endorse and adopt PGx testing as a practice in their day-to-day clinical care regime.

##### 3.2.3.2 Affordability

For young people, the financial impact of PGx testing is a critical hurdle to overcome. Not only do costs impact upon individual participants and their personal concerns with PGx testing, but the financial burden accompanied with the integration of PGx testing into clinical use was perceived as a significant barrier to widespread access amongst youth. Furthermore, participants assumed that PGx testing would be subsidized by Medicare, waiving or reducing the financial burden of testing. When explained by moderators that Medicare currently does not cover PGx testing, participants expressed adamant concerns that PGx testing would be unaffordable for the youth population, stating that PGx testing “*should not be more than $100*”.

Participants emphasized the need for Medicare coverage to include PGx testing to make it a viable option for more young individuals, particularly those from lower-income backgrounds and for those who may not have the support of families to assist them financially.

*“I do not think there will be many people who can afford it.”* [FG3P1]

##### 3.2.3.3 Lack of awareness

Participants noted the lack of awareness and community education around PGx in youth mental healthcare. Only one participant had previously received a PGx test for guided antidepressant treatment, with one other aware of PGx testing, though they had not pursued it. All other participants admitted to a lack of knowledge about PGx testing and its potential benefits for personalized antidepressant treatment.

*“Looking back on it now, I wish I’d had PGx testing as an option so that it could help, because your medication choice, it is going to be with you for a very long time.”* [FG1P1]

Additionally, there was a perceived absence of comprehensive education initiatives within communities to promote an understanding of genetics and its relevance to mental healthcare. Young people believed that by focusing attention on overcoming other barriers, such as GP knowledge and adoption, that youth and broader community awareness would follow suit. One participant even speculated that the addition of PGx testing to the Medicare Benefit Schedule could not only prove beneficial for young people financially, but also increase the awareness of PGx testing availability amongst the community.

*“I feel like if it gets funded by Medicare, it might, probably become common knowledge through, like, media resources as well.”* [FG2P1]

##### 3.2.3.4 Limited accessibility

Many participants thought that geographic disparities in healthcare access could present as a significant barrier to widespread adoption of PGx testing in Western Australia, particularly in rural and remote regions. Participants noted that limited access to testing kits and information about PGx benefits in mental health treatment could exacerbate healthcare inequalities, potentially restricting rural youth from accessing personalized PGx-based therapies.

*“Especially with rural, that might be a bit of a challenge too, not only access to, you know healthcare clinicians, but to see where this test is available in your area.”* [FG2P2]

## 4 Discussion

Overall, our results suggest that incorporating a PGx-guided approach to antidepressant treatment may address the medication efficacy and tolerability concerns of young Australians starting pharmacotherapy for depression and/or anxiety. Our study in young participants naïve to the practice of PGx testing who are still navigating the antidepressant pharmacotherapy on a trial-and-error basis showed a general optimism towards PGx practices, consistent with prior studies (Stancil et al., 2021). Yet, our findings also highlight that integrating PGx into primary mental healthcare to guide antidepressant pharmacotherapy does not come without concerns that need navigating.

Many participants expressed their dissatisfaction with their lack of involvement in the decision-making process for their antidepressant treatment and understand that the use of PGx may assist in involving them further by increasing their engagement with their GP. Furthermore, participants discussed that PGx testing would help them to feel that their treatment choice was personalized, helping them to feel listened to, validated, and understood by their clinician. Previous research has found that young people frequently express a strong desire for autonomy, taking higher levels of responsibilities for their health by being better informed of their options and thereby more involved in the decision-making process (Fleary and Joseph, 2020; Freeman et al., 2018; Freeman et al., 2020). The conversations that took place in the present study demonstrate that these needs are not currently being met in primary mental healthcare practice, with a significant disparity between expected and actual involvement in the treatment decision-making process. While adolescents may benefit from professional support and guidance during medical decision-making processes (Grootens-Wiegers et al., 2017), our study participants primarily expressed a desire for more personalized treatment to fit their unique circumstances. The lack of comprehensive discussions in primary care practice shown in this study echoes the current calls for better communication between healthcare providers and youth patients (Patak et al., 2009). Moreover, prioritizing prescribing practices that consider individualized factors for each patient, such as PGx, need to be explored to empower youth and satisfy their need for personalized care.

With many participants having had poor experiences on past antidepressants, all participants were optimistic about the possibility of PGx testing to increase antidepressant efficacy and tolerability, minimizing medication-related side effects whilst increasing the likelihood of symptom remission. These sentiments and understandings are reciprocated in other populations of young people and are not specific to those treated with antidepressant pharmacotherapy (Stancil et al., 2021). Previous literature demonstrates the difficulty in managing antidepressant pharmacotherapy in young people (Safer and Zito, 2019; Spielmans and Gerwig, 2014). Compared to placebo, few antidepressants have shown a significant therapeutic effect in treating the symptoms of major depressive disorder in young people when compared to their adult counterparts (Cipriani et al., 2016; Zhou et al., 2020). Further still, young people present a significantly different adverse-drug reaction profile than adults, with a higher rate of incidence in developing medication-related side effects (Liu et al., 2019). These challenges were observed in our participant cohort with almost two-thirds of participants failing their initial treatment and beginning a process of medication trial-and-error. Considering this medication fail rate even in a small sample size, this study supports the need for more effective antidepressant prescription methods in young people. Though PGx-guided antidepressant pharmacotherapy is suggested to play a significant role in improving patient outcomes in youth psychiatric care (Roberts et al., 2023), as echoed in the concerns of our youth participants, more age-appropriate randomized controlled trials are required to demonstrate the efficacy for PGx testing to successfully prescribe more effective and tolerable antidepressants to young people specifically.

The overwhelming positivity towards the integration of PGx into clinical use did not come without participant concerns. Apprehensions around the perceived high costs, delays in obtaining results and accessing treatment, and privacy issues related to genetic data handling were raised in our focus group and interview sessions, reflecting broader uncertainties in the field (Joly et al., 2014; Young and MacDougall, 2023). The perceived-financial burden of PGx testing was of utmost concern. This concern has been noted across age groups and populations, as well as professions, with patients, clinicians, and pharmacists all perceiving cost as a key barrier to the integration of PGx into clinical use (Liko et al., 2020; Chan et al., 2017; Shishko et al., 2015; Dunbar et al., 2012; Laplace et al., 2021). Furthermore, healthcare providers note the delay period in waiting for PGx testing results as a barrier to use (Vest et al., 2020; Dunbar et al., 2012; Laplace et al., 2021). Participants expressed their concerns that costs exceeding 100 AUD and wait times of more than 1 week would be too much to ask of young people who need immediate treatment for depression or anxiety. Given that current PGx testing processes cost on average 180 AUD and take approximately 10 business days (Forbes et al., 2023), improvements to these services will need to be made to increase the likelihood of youth engagement in PGx.

Youth-perceived barriers identified in this study include a perceived lack of awareness and education about PGx testing among both healthcare providers and the public, financial constraints due to high testing costs, and social and geographical limitations to testing access. These youth-perceived barriers align with those expressed by adults in the literature (Jameson et al., 2021; Pinzón-Espinosa et al., 2022; Virelli et al., 2021). Concerns about healthcare providers readiness to uptake PGx testing into their care practice is echoed across the literature, with numerous healthcare providers expressing their lack of understanding in using PGx testing (Shishko et al., 2015; Chan et al., 2020), as well as their lack of time to include PGx reporting into their clinical workflow and consultation times (Shishko et al., 2015; Vest et al., 2020; Dunbar et al., 2012; Goodspeed et al., 2019; Ferwerda et al., 2024). Youth being unaware of PGx testing as an available option for informed prescription presents as a significant barrier to successfully integrating PGx testing into clinical care. However, many young people expect to hear about the treatment options, such as PGx testing, through their clinicians, and it is these primary care clinicians who are perceived as being unaware of the utility of PGx testing. Efforts need to be made to increase the community understanding and awareness of PGx testing through provider and consumer education platforms (Omran et al., 2023; Hayward et al., 2021; Mai et al., 2024; Frigon et al., 2019). Additionally, Australia is a geographically large nation with over 30% of the nation’s people living outside of metropolitan areas, and thus, PGx needs to be made equitably accessible in regional and rural environments. Several PGx testing companies in Australia utilize a mail service model that navigates this issue. However, it is important that randomized controlled trials include a geographically diverse sample population to ensure the benefits of PGx-guided treatment are observed outside of metropolitan regions.

Further emphasized as not just individual participant concerns but as youth wide perceived barriers was the costing of genetic testing. Young, tertiary-aged students are already navigating significant financial hurdles, and thus, to interact with genetic health services such as PGx, the cost must be attainable, which many of our study participants believed it not to be. Yet to obtain have PGx services added to the Medicare Benefit Schedule and lower the out-of-pocket expenses for patients, further randomized controlled trials are needed to demonstrate not only the effectiveness of PGx-guided antidepressant treatment, but as well the accessibility. As mentioned by a participant of this study, with further trial evidence supporting the efficacy of PGx-guided antidepressant pharmacotherapy and an increased awareness and demand for personalized mental healthcare, policy reform to subsidize the financial burden of PGx testing may be amenable. Though recent trials have aimed to determine the efficacy and tolerability of PGx-guided antidepressant treatment in young people, the effectiveness of such treatment remains nuanced and further work is required to understand the impact of PGx testing in youth mental health before youth concerns and perceived-barriers can be navigated and accounted for (Nooraeen et al., 2024; Namerow et al., 2022; Voort et al., 2022).

There are some limitations to the present study. Participants were recruited predominantly through advertisement on university campuses; thus, our findings may not be reflective of the concerns and perceived barriers of young adults who have not pursued tertiary education. Furthermore, the gender representation in our sample is female biased and the concerns of males receiving antidepressant treatment may be underrepresented in our findings. This is not surprising, however, considering the low rate of help-seeking in young males suffering mental health conditions and the higher rate of mental illness in adolescent and young adult females (Australian Bureau of Statistics, 2022). While participants received an explanation of PGx testing and its role in guiding antidepressant prescriptions, and session moderators addressed questions regarding PGx logistics, no formal assessment of their understanding or knowledge was conducted. Future qualitative studies exploring youth perspectives on PGx testing should include a knowledge assessment to ensure participants have a clear understanding of the concepts before sharing their views. Lastly, this study was conducted in Western Australia, which may have specific healthcare practices and cultural attitudes that differ from other regions, affecting the applicability of the findings to other contexts.

In conclusion, this study highlights the unique perspectives and concerns of young adults towards the implementation of PGx testing to optimize and guide antidepressant pharmacotherapy. Future studies must continue to employ a holistic approach, prioritizing these concerns when considering best practice for the treatment of mental health in young people. Whilst further randomized controlled trials are needed, future trials must look beyond measures of efficacy and tolerability, assessing patient satisfaction, cost-effectiveness, and treatment adherence in relation to PGx-guided treatment antidepressant treatment. This breadth of understanding will allow policymakers, clinicians, and researchers to gain a broader perspective of the holistic impact of PGx testing to determine whether Medicare subsidy is appropriate for all PGx services. Understanding the concerns of youth identifies key outcome measures for future studies, ensuring that trial procedures and findings answer the questions and queries raised by young people. Furthermore, fundamental resources to educate and inform the community on PGx practices *need* to be prioritized. The evidence for PGx to improve the efficacy and tolerability of pharmacotherapy is readily available, yet fundamental barriers inhibit its uptake into clinical practice. To improve and maintain the therapeutic relationship, young people need to see that their clinicians are equipped with the knowledge to successfully use PGx o guide antidepressant treatment. Prior research highlights a self-reported gap among healthcare providers in understanding and integrating PGx into clinical practice. To address this, educational platforms tailored to clinicians’ must be developed, equipping them with the tools necessary for effective and widespread implementation. By addressing these concerns and barriers in future research, healthcare providers and researchers can work towards a more personalized and effective approach to antidepressant pharmacotherapy, ultimately improving outcomes for young adults struggling with mental health issues.

## Data Availability

The original contributions presented in the study are included in the article/Supplementary Material, further inquiries can be directed to the corresponding authors.
