# Corrigendum: Exploring perceived barriers and attitudes in young adults towards antidepressant pharmacotherapy, including the implementation of pharmacogenetic testing to optimize prescription practices

**DOI:** 10.3389/fphar.2025.1590955

**Published:** 2025-03-26

**Authors:** Bradley Roberts, Zahra Cooper, Georgia Landery, Susanne Stanley, Bernadette T. Majda, Khan R. L. Collins, P. Anthony Akkari, Sean D. Hood, Jennifer Rodger

**Affiliations:** ^1^ Perron Institute for Neurological and Translational Science, Nedlands, WA, Australia; ^2^ School of Biological Sciences, The University of Western Australia, Crawley, WA, Australia; ^3^ School of Biomedical Sciences, The University of Western Australia, Crawley, WA, Australia; ^4^ Division of Psychiatry, School of Medicine, The University of Western Australia, Crawley, WA, Australia; ^5^ Curtin Medical School, Curtin University, Bentley, WA, Australia; ^6^ North Metropolitan Health Service, Western Australian Department of Health, Nedlands, WA, Australia; ^7^ School of Human Sciences, The University of Western Australia, Crawley, WA, Australia; ^8^ Centre for Molecular Medicine and Innovative Therapeutics, Murdoch University, Murdoch, WA, Australia; ^9^ Division of Neurology, Duke University Medical Centre, Duke University, Durham, NC, United States

**Keywords:** pharmacogenetics, antidepressant pharmacotherapy, depression and anxiety, youth, young adults, community perspective, clinical implementation

In the published article, there was an error. A general nation-wide statistic has been stated incorrectly during changes made in the review process. Whilst the grammatical/typo error is minor in nature, this correction is necessary to stop misinformation and improper citations.

A correction has been made to **1. Introduction**, Paragraph 2. This sentence previously stated:

“The Australian prescription rate of antidepressants has risen by ∼25% per annum over the past 7 years (Australian Institute of Health and Welfare, 2023; Australian Institute of Health and Welfare, 2022; Australian Institute of Health and Welfare, 2021a; Australian Institute of Health and Welfare, 2020; Australian Institute of Health and Welfare, 2019; Australian Institute of Health and Welfare, 2018), with over 33 million antidepressants prescribed in 2023 alone (Australian Institute of Health and Welfare, 2023).”

The corrected sentence appears below:

“The Australian prescription rate of antidepressants has risen by ∼33% over the past 7 years (Australian Institute of Health and Welfare, 2023; Australian Institute of Health and Welfare, 2022; Australian Institute of Health and Welfare, 2021a; Australian Institute of Health and Welfare, 2020; Australian Institute of Health and Welfare, 2019; Australian Institute of Health and Welfare, 2018), with over 33 million antidepressants prescribed in 2023 alone (Australian Institute of Health and Welfare, 2023).”

The authors apologize for this error and state that this does not change the scientific conclusions of the article in any way. The original article has been updated.

