# Supplementary material for: Exploring perceived barriers and attitudes in young adults towards antidepressant pharmacotherapy, including the implementation of pharmacogenetic testing to optimize prescription practices
Source: Front Pharmacol. 2025 Jan 3;15:1526101. doi: 10.3389/fphar.2024.1526101 (PMC11739104; doi:10.3389/fphar.2024.1526101)
Supplement: Supplementary file 1 [file DataSheet1.docx]

Supplementary Material

# Supplementary Material 1. Reflexive Thematic Analysis Process

**Overview**

This study employed Reflexive Thematic Analysis (RTA) following the framework outlined by Braun and Clarke (2024). The analysis was conducted within an interpretivist paradigm, aiming to explore and interpret participants’ subjective experiences and perceptions related to antidepressant pharmacotherapy and pharmacogenetic testing.

**Epistemology**

The study adopted an interpretivist approach, acknowledging that participants’ experiences are inherently subjective and shaped by their cultural, social, and individual contexts. Themes were constructed to reflect both the explicit meanings expressed by participants and the broader social and psychological dynamics underlying their experiences. The researchers recognize their active role in interpreting participants' narratives, with themes shaped through their interpretative lenses.

| **Epistemology** | Interpretivist |
| --- | --- |
| **Emphasis** | Understanding participants’ experiences |
| **Reality** | Subjective meanings shaped by context |
| **Researcher Role** | Interpreter of participants’ experiences |

**Reflexive Thematic Analysis Steps**

1. **Familiarisation with the Data**

All focus group recordings were transcribed verbatim.

The research team immersed themselves in the data by reading and re-reading the transcripts to develop an in-depth understanding of participants’ narratives.

Initial notes and impressions were documented to capture potential areas of focus.

1. **Coding**

The primary coder, present during all focus groups, conducted the first round of coding.

Open codes were created first followed by descriptive codes.

Descriptive codes were applied inductively (emerging from the data) and deductively (guided by the study’s aims and framework).

The process accounted for both semantic (explicit) and latent (implicit) meanings in the data.

1. **Collaboration in Coding**

A second coder, external to the research team and within the participants’ age demographic (18-24 years), independently coded the transcripts.

This ensured that codes were consistent and that alternative interpretations of the data were considered.

Codes were reviewed by a Reflexive Moderator and any discrepancies or differing interpretations were discussed and reconciled through team discussions.

1. **Theme Development**

Codes were grouped into potential themes through iterative discussions among the research team.

The involvement of the age-matched researcher provided contextual insights into generational and cultural nuances, while the senior researchers contributed an external lens to challenge assumptions.

Themes were reviewed and refined collaboratively to ensure coherence and clarity.

1. **Defining and Naming Themes**

Each theme was defined to encapsulate its core meaning and relationship to the research questions.

Themes were named to reflect the participants’ language and experiences wherever possible.

1. **Finalisation and Reporting**

Supporting data extracts (quotes) were selected to illustrate each theme.

Themes were woven into a narrative that aligned with the study’s aims and highlighted participants’ perspectives.

**Reflexivity**

The research team engaged in reflexive practices to ensure that the analysis was grounded in the data while acknowledging the influence of researchers’ positionalities.

- **Team Discussions**: Regular meetings provided a space to critically examine individual interpretations and ensure that the final themes reflected participants’ views rather than researchers’ biases.
- **Audit Trail**: A detailed record of coding decisions, theme development, and iterative revisions was maintained for transparency.

**Contributions of the Research Team**

1. **Primary Coder (First Author)**

The primary coder was present as the facilitator or co-facilitator in all focus groups and directly interacted with participants during data collection. This involvement provided a deep familiarity with the data and enriched the analytical process by allowing the coder to integrate observational insights from the group dynamics into the thematic development.

His role ensured continuity between data collection and analysis, fostering an in-depth understanding of the context and nuances of participants' responses. The primary coder maintained regular discussions with the second coder and reflexive moderator to critically examine how their interpretations might be influenced by their interactions with participants or personal perspectives on the topic.

- **Role in Theme Development**: The primary coder ensured the themes captured the depth of participants’ narratives while continuously reflecting on whether their facilitation role shaped the data collection or interpretation in unintended ways.

1. **Second Coder (External to the Team)**

The second coder was external to the research team and did not participate in data collection. As an individual within the participants’ age group (18–24 years), the second coder brought a valuable insider understanding of the social and cultural contexts shaping participants’ experiences. Her shared generational perspective helped identify subtle but meaningful elements in the data that might have been overlooked by older researchers. Her independent analysis helped to validate and expand the primary coder’s interpretations, ensuring the themes captured the breadth of the data. The second coder reflected on their own potential biases as someone external to the study design and data collection process. For instance, they considered whether their own experiences as a member of the participants’ demographic influenced their interpretations. During coding discussions, they critically engaged with the primary coder and reflexive moderator to ensure their interpretations were based on the data rather than assumptions tied to their shared demographic.

- **Role in Validation:** The second coder’s independent analysis served as a check against potential biases introduced by the primary coder’s direct interaction with participants.

This reflexive engagement helped ensure that the themes captured diverse interpretations and perspectives within the data.

1. **Reflexive Moderator (Study Coordinator)**

The study coordinator moderated discussions between the primary and second coder, ensuring that diverse perspectives were integrated into the analysis. Her experience in thematic analysis provided guidance on refining themes and maintaining alignment with the study’s aims and interpretivist framework. She critically examined potential biases introduced by the primary coder’s dual role as a facilitator and analyst, promoting reflexivity and methodological rigour.

**Role in Ensuring Rigour:** By facilitating discussions on discrepancies and encouraging iterative refinement of themes, the reflexive moderator ensured that the final thematic framework reflected a balance of emic (insider) and etic (outsider) perspectives.

Their oversight helped maintain consistency with the interpretivist paradigm, ensuring that participants’ voices remained central to the analysis.

**Collaborative Reflexivity**

Reflexivity was not limited to individual roles but was actively practiced through collaboration:

- Team members engaged in regular reflective discussions to explore how their diverse roles and perspectives shaped the analysis.
- All decisions regarding coding, theme development, and narrative construction were documented and critically examined to ensure transparency and accountability.
- The iterative dialogue between team members allowed for a richer, more nuanced understanding of the data, ensuring that the themes represented both individual and collective interpretations.

**Additional Notes**

**Ethical Considerations:** Confidentiality and anonymity of all participants were maintained during the RTA process. Each participant was given an identifier (consisting of the focus group session the attended, and a participant number that was assigned to them during the session, e.g. participant 1 in the 1^st^ focus group: FG1P1

**Supplementary Resources:** All coding matrices and theme development tables are available for review.
